# Valorization of wine industry by-products as a flavoring agent in water kefir: microbiological viability and functional properties

**DOI:** 10.3389/fnut.2026.1856957

**Published:** 2026-06-11

**Authors:** Ana Carolina Mendes Oliveira Campos, Ana Elisa Barbosa Siqueira, Maria Isabela da Silva Figueiredo, Marcos Antônio Soares, Claudia Puerari, Maressa Caldeira Morzelle, Julio Cesar Colivet Briceno, Juliana Aparecida Correia Bento

**Affiliations:** 1 Food and Nutrition Department, Faculty of Nutrition, Federal University of Mato Grosso (UFMT), Cuiabá, Brazil; 2 Post-graduate Program in Tropical Agriculture, Federal University of Mato Grosso (UFMT), Cuiabá, Brazil; 3 Laboratory of Biotechnology and Microbial Ecology, Institute of Biosciences, Federal University of Mato Grosso, Cuiabá, Brazil

**Keywords:** agro-industrial by-products, *Caenorhabditis elegans*, functional beverage, grape pomace, sensorial acceptance

## Abstract

**Introduction:**

This study developed a functional water kefir beverage flavored with grape pomace (*Vitis labrusca*.) and evaluated its physicochemical, microbiological, and sensory properties, alongside its functional effects in a *Caenorhabditis elegans* model.

**Methods:**

Four treatments were tested: 0% (KC), 25% (K25), 37.5% (K37.5), and 50% (K50) pomace addition.

**Results:**

Anthocyanin and phenolic contents were dose-dependent, peaking in K50 (33.56 mg L^−1^ and 4.31 mg g^−1^, respectively). During 21 days of refrigerated storage, a decrease in reducing sugars, pH (4.3 to 3.5), and a concomitant increase in acidity (up to 6.60%) were observed, reflecting continuous microbial metabolic activity. Due to excessive acidity and residual taste, K37.5 and K50 were deemed unfeasible. Consequently, K25 was selected for biological and sensory validation. In K25, yeast and acetic acid bacteria (AAB) maintained high viability (10^7^ CFU mL^−1^), whereas lactic acid bacteria (LAB) remained below 10^5^ CFU mL^−1^ due to the selective pressure of the grape pomace. K25 achieved a sensory acceptance index of 76.11% and a 65% purchase intention.

**Discussion:**

These results indicate that water kefir flavored with 25% grape pomace is a viable probiotic carrier rich in bioactive compounds with promising sensory appeal.

## 1 Introduction

Grape pomace (GP) is a significant residual byproduct of the juice and wine industries, composed of seeds, skins, and pulp remaining after extraction. The importance of its utilization lies in environmental waste management, as considerable volumes of material are generated annually; in 2023, it was estimated that Brazil produced 378,319 tons of solid grape waste, a large portion of which lacked proper disposal (1). The central importance of this research lies in the valorization of a byproduct (grape pomace) that is often discarded as waste. Reusing this residue as a raw material for a new product, rather than discarding it, represents an economically viable alternative and enhances sustainability (2, 3). This aligns with global trends favoring “natural, functional, and clean label products” and addressing resource efficiency and waste reduction. Thus, the reuse of this waste aligns with the UN Sustainable Development Goals (SDGs), particularly those related to achieving a circular economy and zero waste. This approach involves integrated production and the participation of local communities in waste-free production organizations.

Martins et al. (1) developed and characterized fermented milk with different concentrations of *Vitis labrusca* grape pomace (GP) and investigated its effect on the physicochemical, microbiological, and sensory characteristics of the formulations. The study found that incorporating GP prior to fermentation (method F1) was the most appropriate technological approach, as it improved water retention capacity (WRC) and reduced syneresis, resulting in greater structural stability. Regarding bioactivity, formulation F1 (with 25% of GP w/w) resulted in a 900% increase in total phenolic content (TPC) compared to the control, and a significant increase in antioxidant activity. Lactic acid bacteria (LAB) in all samples remained viable during storage, with counts exceeding 10^7^ CFU/mL. Finally, formulation F1 with 12.5% GP achieved the highest sensory acceptance score (9.0), demonstrating the best balance between technological advantages and consumer preference.

Another avenue not yet explored in the literature is the use of grape pomace to produce a plant-based kefir-like beverage. The use of fruit byproducts, specifically pomace, to produce water kefir (also referred to as water kefir-like beverages or WKB) is justified by the increasing consumer preference for non-dairy fermented beverages, which appeal to vegans, vegetarians, and individuals with lactose intolerance or dairy allergies (4, 5). Unlike industrial fermentations that utilize defined monocultures, the water kefir consortium—comprising heterogeneous populations of lactic acid bacteria, acetic acid bacteria, and yeasts—is highly sensitive to variations in scale, mass transfer, and shear stress within bioreactors. These fluctuations can induce metabolic shifts over consecutive fermentation cycles, altering the production of organic acids, volatile aromatic compounds, and exopolysaccharides, which directly compromises batch-to-batch reproducibility. Furthermore, incorporating agro-industrial by-products introduces seasonal raw material variability, affecting fermentation kinetics and substrate composition. Controlling the fermentation endpoint to prevent post-bottling acidification and secondary carbon dioxide accumulation, while simultaneously maintaining probiotic cell viability (10^6^–10^7^ CFU/mL) without resorting to thermal pasteurization remains a critical bottleneck for ensuring the shelf-life stability and sensory standardization of the commercial beverage.

Fermenting fruit juices or extracts is a strategy to minimize post-harvest losses and introduce new, health-enhancing products (2, 3, 6). Across wine industries, grape pomace is dominated by polyphenols (anthocyanins, catechins/procyanidins, flavanols, phenolic acids, stilbenes) (7). Frequently highlighted individual compounds include catechin, epicatechin, procyanidins (proanthocyanidins), quercetin, myricetin, kaempferol, gallic acid, caffeic acid, p-coumaric acid, resveratrol, and anthocyanins such as malvidin, petunidin, cyanidin, peonidin, delphinidin. Additionally, a substantial dietary fiber is mentioned and, in the seed fraction, lipids rich in linoleic acid and vitamin E. Exact profiles vary with grape variety and vinification, but these groups consistently represent the most predominant compounds (8). Also, grape pomace obtained after different technological approaches during the fermentation showed as a rich source of phenolic compounds (9).

The justification for focusing on grape pomace specifically stems from its rich composition; similar fruit residues, such as *Aronia melanocarpa* pomace, have been shown to contain considerable quantities of phenolic compounds, which are often retained in plant byproducts. These compounds can contribute to the final product’s functional properties, such as antioxidant activity (10). Moreover, producing WKB addresses the rising global demand for probiotic foods, a market projected to reach $ 2.19 billion by 2026 (2, 10). However, industrial-scale production of WKB, particularly with unconventional substrates, such as grape pomace, faces challenges regarding the need for reproducible products with standardized composition and quality. Thus, the evaluation of the physicochemical and microbiological stability during storage directly addresses a significant knowledge gap necessary for commercial viability, as stability and microbial viability must be ensured throughout the product’s shelf life to maintain probiotic functionality.

The functional evaluation of foods containing prebiotics and probiotics often relies on animal models, but this approach is limited by ethical considerations regarding the use of animals in research, in addition to the high costs associated with the breeding, maintenance, and handling of these biological models (11, 12). The model nematode *Caenorhabditis elegans* has proven to be an effective alternative system for assessing food functionalities, such as antioxidant activity, effects on longevity, and regulation of lipid homeostasis (13, 14, 15).

Given this, we hypothesize that: the grape pomace, due to its remaining fermentable compounds and high concentration of bioactive substances (e.g., polyphenols), will successfully serve as a substrate for water kefir grains, resulting in a viable, non-dairy probiotic beverage that exhibits enhanced physicochemical stability and retains adequate levels of probiotic microorganisms (above 7 log CFU/mL for LAB) throughout the storage period. This study aimed to develop a water kefir beverage flavored with wine industry by-products. Furthermore, it sought to evaluate its physicochemical characteristics and microbiological viability during storage, as well as to assess its functional properties using a *Caenorhabditis elegans* model and sensory acceptance.

## 2 Materials and methods

### 2.1 Material and reagents

Grape pomace (*Vitis labrusca*), Pinot Noir pomace, was obtained after the initial stage of wine production and supplied by Locanda do Vale winery, located in the municipality of Chapada dos Guimarães, Mato Grosso, Brazil (15.4643° S, 55.7495° W). The soils of the Chapada dos Guimarães region (MT) are predominantly Latosols, Quartzarenic Neosols, and soils of sandy origin. They are characterized by high porosity (ideal for water absorption), but also by their high acidity and low natural fertility. The vegetative period for the vines coincides with a transition from the rainy season to the drier, milder season. Thus, in April there is rainfall (average of 119 mm) and temperatures vary between 21 °C (minimum) and 30 °C (maximum), with hot days and cooler nights. In the following months, with scarce rainfall (12 mm), temperatures ranged between 18 °C and 29 °C in the region. To completely prevent biomass degradation and the development of mycotoxigenic fungi (such as *Aspergillus* or *Penicillium strains*) during storage, the grape pomace samples were collected immediately after the winemaking process and promptly frozen at –20 °C until the moment of use. The composition of the grape pomace used was moisture: 70.2 g/100 g; ash: 2.12 g/100 g; protein: 2.43 g/100 g; lipids: 7.49 g/100 g; total dietary fiber: 9.77 g/100 g; carbohydrates: 7.99 g/100 g. The water kefir grains (a natural, complex, and variable symbiotic consortium of microorganisms embedded in a polysaccharide matrix) used is maintained by the FermentLab at the Faculty of Nutrition (FANUT), Federal University of Mato Grosso (UFMT).

The chemical standards for phenolic compounds and monomeric anthocyanins, including gallic acid, as well as the stable free radical 2,2-diphenyl-1-picrylhydrazyl (DPPH) used for antioxidant activity assays, and Folin–Ciocalteu were purchased from Sigma-Aldrich (St. Louis, MO, United States). Microbiological culture media, including De Man, Rogosa and Sharpe (MRS) agar, M17 agar, and Potato Dextrose Agar (PDA), were obtained from Difco Laboratories (Detroit, MI, United States). All other reagents used were analytical grade, purchased locally.

### 2.2 Water kefir preparation

After preliminary tests to adjust the fermentation conditions, the study design was established. The experiment consisted of four treatments, in duplicate: a control (no residue), and additions of 25.0 (K25), 37.5 (K37.5), and 50.0% (K50) grape pomace residue extract (v/v). Grape pomace residue extract was homogenized using the Ultra Turrax T25 digital model (IKA, Staufen, Germany) for 15 min, 15,000 rpm, yielding an extract containing 60% water and 40% pomace (w/w), pasteurized at 80 °C for 5 min and sieved. This thermal treatment served a dual purpose: first, it ensured strict microbiological safety by eliminating any competitive native microbiota from the winery environment; second, the thermal process facilitated the volatilization and complete removal of any residual ethanol originating from the primary winemaking fermentation, preventing unexpected alcohol spikes in the final beverage.

Kefir beverage was prepared as described by Puerari et al. (2012). The fermentation was carried out in 1000 mL glass vessels, with a working volume of 800 mL per batch. The treatments were inoculated with 8% brown sugar and 5% water kefir grains (w/v). First fermentation was carried out under aerobic conditions at 25 °C for 24 h. The second fermentation occurred anaerobically at 25 °C for 12 h to naturally carbonate the beverage. After fermentation, the beverages were stored at 2–4 °C until analysis, to slow the growth of microorganisms and ensure the stabilization of the beverage.

The study was divided into three stages: in the first stage, physicochemical analyses, total phenolic compounds content, monomeric anthocyanins, and antioxidant activity of the beverages were performed; in a second moment, aliquots of the beverages were withdrawn during refrigerated storage for the evaluation of the physicochemical stability of the beverage on days 1, 7, 14, and 21 of storage. Analyses of phenolic compounds and monomeric anthocyanins were also carried out throughout storage, on days 1, 10, and 21. The final stage consisted of evaluating the microbiological stability and functionality of the beverage that exhibited the best physicochemical stability in the previous step. Additionally, a sensory acceptance test was conducted.

### 2.3 Physic-chemical evaluation of beverage constituents

Total solids content (AOAC 925.45) was determined in an oven at 105 °C. Ash (AOAC 923.03) was quantified by incineration in a muffle furnace. All the methods followed those proposed by AOAC (16) and were carried out in triplicate. Measurements of pH were determined electrometrically using a digital pH meter (Tecnopon, mpa-210, Brazil) previously calibrated with pH 4.0 and 7.0 buffer solutions (17). Soluble solid content (SSC), reported as °Brix, was measured using Abbé refractometer. Total titratable acidity (TTA) was determined by titration of the samples with 0.1 N NaOH to an end point of pH 8.2–8.4 as described in ISO 11869:2012 and expressed as g/L of total acid.

### 2.4 Reductor sugar

The determination of reducing sugars was carried out using the Somogyi–Nelson method, following the protocol described by Maldonade et al. (18). The absorbance of the developed color was measured at 540 nm using a spectrophotometer, and quantification was performed by comparison with a glucose standard curve. Results were expressed as milligrams of glucose equivalent per gram of dry sample (mg/g).

### 2.5 Determination of monomeric anthocyanins

The determination of monomeric anthocyanins was performed according to the method described by Lee et al. (19). Briefly, the appropriate dilution factor of the sample with pH 1.0 buffer was determined until the absorbance at 520 nm was between 0.2 and 1.4. The same dilution factor was used to prepare two dilutions with a pH 4.5 buffer. The absorbance of the samples diluted in the buffers was then determined at 520 and 700 nm. The concentration of anthocyanin pigments, expressed as cyanidin-3-glycoside equivalents, was calculated according to Equation 1.


$$\begin{array}{l} \text{Cianidin}-3-\text{glucoside equivalents}\;(\mathrm{mg}/L)\\ =\frac{A\times \mathrm{MW}\times \mathrm{DF}\times 10^{3}}{\varepsilon \times 1} \end{array}$$ (1)

Where: A = (A520nm – A700nm) at pH 1.0 – (A520nm – A700nm) at pH 4.5; MW (molecular weight) = 449.2 g/mol for cyanidin-3-glycoside; DF = established dilution factor; 1 = optical path length (spectrophotometer cuvette); ɛ = 26,900, molar coefficient, for cyanidin-3-glycoside; 10^3^ = conversion factor from g to mg.

The analysis was performed in triplicate on samples stored on days 1, 10, and 21.

### 2.6 Total phenolic compounds

Total phenolic compound (TPC) contents were determined using the Folin–Ciocalteu method, as described by Singleton et al. (20). Absorbance was measured at 765 nm with a spectrophotometer (Biochrom, Libra S32, Cambridge, United Kingdom) and analyzed in quadruplicate. A calibration curve was constructed using gallic acid as the standard, and the results were expressed as milligrams of gallic acid equivalents per mL of kefir (mg GAE g^−1^).

### 2.7 Antioxidant activity

The antioxidant potential was assessed using the DPPH (2,2-diphenyl-1-picrylhydrazyl) method, as described by Brand-Williams et al. (21). The Trolox equivalent antioxidant activity of the DPPH radical was quantified spectrophotometrically at 517 nm (Biochrom, Libra S32, Cambridge, United Kingdom) after a 30-min reaction period. Antioxidant activity was determined from a standard Trolox curve, with results expressed as μM of Trolox mL^−1^ sample.

Furthermore, the ferric reducing antioxidant power (FRAP) was estimated following the methodology reported by Pulido et al. (22). For this, 90 μL of the samples were transferred to tubes containing 270 μL of distilled water and 2.7 mL of FRAP reagent. Samples were homogenized using a tube shaker (Phoenix-AP56, São Paulo, Brazil) and subsequently incubated in a water bath (572-Fisatam, São Paulo, Brazil) at 37 °C. After 30 min, absorbance at 595 nm (Biochrom, Libra S32, Cambridge, United Kingdom) was analyzed in quadruplicate, and results were expressed as μmol ferrous sulfate equivalent mL^−1^ dry sample.

### 2.8 Functionality in an *in vivo* model

#### 2.8.1 Strains and culture of *Caenorhabditis elegans*

The *C. elegans* N2 (wild-type) strain and BA17 [fem-1(hc17) IV] were maintained at 15 °C on plates containing Nematode Growth Medium (NGM) 2.5 g/L peptone, 3 g/L NaCl, 15 g/L bacteriological agar, 1 M MgSO. H_2_O, 1 M CaCl_2_, 5 mg/mL cholesterol, 1 M KH_2_PO_4_, using *Escherichia coli* OP50 as food, previously grown in Luria-Bertani (LB) broth (10 g/L peptone, 5 g/L yeast extract, 5 g/L NaCl). The population of *C. elegans* N2 was synchronized to obtain worms at the L4 stage (23).

#### 2.8.2 Beverage preparation for *in vivo* assays

The beverage containing 25% of fermented grape pomace (K25) was centrifuged at 5,000 rpm for 5 min, and subsequently, the supernatant was sterile filtered using a 0.22 μm membrane filter Millipore®. We added 200 μL of the sterile solution to the NGM plates, and then each plate was inoculated with 100 μL of *E. coli* OP50 previously inactivated by autoclaving (121 °C, 15 min).

#### 2.8.3 Evaluation of oxidative and thermal stress reduction

We assessed the ability of the K25 beverage to reduce oxidative stress in *C. elegans* using worms at the L1 stage. The worms were treated with the K25 beverage at 20 °C until they reached the L4 stage. Following the treatment, adult worms (30 worms per group) were added to 48-well plates containing liquid NGM, 3 mM H_2_O_2_ to induce oxidative stress, and K25. The number of live and dead animals was evaluated every 2 h (24). The thermal stress assay was conducted according to the previously reported method. After the treatment, the worms were transferred to 48-well plates for thermal stress evaluation. The number of dead worms was recorded every 2 h following a 35 °C heat shock for 1 h (24).

#### 2.8.4 Longevity assay

The mutant *C. elegans* strain BA17 was used to evaluate the effect of the K25 beverage on nematode lifespan extension, avoiding progeny interference (24). Longevity assays were performed in 96-well plates using liquid K medium, following the protocol described by Kumaree *et al,* (25), with minor modifications. Approximately 30 synchronized L4-stage worms were transferred to wells containing 70 μL of filter-sterilized K25 beverage, Control Kefir (KC), or K medium alone (control). Survival was monitored every 2 days, and worms were scored dead if they exhibited no movement after mechanical stimulation. The assay was conducted in triplicate.

#### 2.8.5 Fat quantification

Fat quantification by Oil Red O staining was performed according to Stuhr et al. (26). Briefly, the *C. elegans* population was treated on NGM containing 200 μL of the K25 beverage. For neutral lipid quantification, the treated worms were washed in PBST and fixed in 40% isopropanol for 3 min. Staining was performed using an Oil Red O solution (0.5 mg/mL diluted in 40% isopropanol) for 2 h in the dark. After washing in PBST (30 min), the animals were mounted on a slide and analyzed using a fluorescence microscope. Images were acquired under 10 × magnification. Fluorescence quantification was performed using ImageJ by calculating the Corrected Total Cell Fluorescence (CTCF), which involves subtracting the image background. The values were normalized by the control group for each experiment.

### 2.9 Microbial viability

For acetic acid bacteria (AAB) counts, the sample was spread-plated in Acetic acid medium (AAM) agar added with nystatin (4000UI mL−1), incubated aerobically at 28 °C for 7 days (27). For lactic acid bacteria (LAB) counts, Man, Rogosa and Sharpe (MRS) agar was added with nystatin (4000UI mL−1) and incubated under anaerobic conditions at 30 °C for 72 h (28). Sabouraud agar added with chloramphenicol (500 mg L−1), incubated under aerobic conditions at 30 °C/120 h, was used for yeast counts (28). The results were obtained as logarithms of the number of colony-forming units mL−1 (log CFU mL−1). The analyses were carried out in duplicate on days 1, 7, 14, and 21 of storage.

### 2.10 Sensorial acceptance

Microbiological safety was verified prior to sensory analysis, including assays for *Salmonella*/25 mL (29), *Escherichia coli* mL^−1^ (30), and Molds and Yeasts mL−1 (26). The results were then compared against Brazilian standards established by Normative Instruction No. 161, dated July 1, 2022 (31). The sensory analysis of the kefir involved affective acceptance tests, utilizing a 9-point hedonic scale, followed by a purchase intention test on a 5-point hedonic scale (32). Volunteer tasters (*n* = 120), aged between 18 and 60 years, comprised both sexes (62% female, 38% male). Tests were conducted in the Sensory Analysis Laboratory at FANUT/UFMT, within isolated booths under white lighting, after the signing of an informed consent form. During the evaluation, participants received coded product formulations (three-digit numbers) in approximately 15 mL portions at 4 ± 2 °C, accompanied by a glass of water. Evaluated attributes included overall acceptability, taste, flavor, and appearance. The Acceptability Index (AI) for each product was calculated according to Equation 2.


$$\mathrm{AI}(\%)=(\frac{A}{B})\times 100$$ (2)

Where A represents the average score obtained for the product, and B signifies the maximum possible score on the evaluation scale.

### 2.11 Statistical analysis

Data underwent normality assessment (Levene test), followed by ANOVA, regression analysis, and Principal Component Analysis (PCA) to determine the effects of concentration and storage time. The PCA was constructed based on Pearson’s coefficient. Tukey’s test (*p* < 0.05) was used for mean comparisons. Statistical evaluation was performed using XLSTAT software.

## 3 Results and discussion

### 3.1 Physicochemical evaluation, phenolic compounds, and antioxidant activity

Table 1 presents the physicochemical evaluation of water kefir beverages, including different treatments with the addition of grape residue (25, 37.5, and 50%) and a control, without residue. The parameters analyzed are moisture, ashes, pH, total acidity, and sugar content (Brix). The addition of grape pomace to the formulation of water-based kefir increased the concentration of total solids and the fixed mineral residue content of the beverage, with a consequent reduction in moisture content. These results are consistent with the literature, which hypothesizes that the introduction of fruit pulps, pomaces, or juices enriches the beverage with all their natural components, including sugars (which increase soluble solids), proteins and fibers (which increase total solids), and a variety of minerals (which increase fixed mineral residue) (33). The presence of these components in the fruit pomace also justifies the results observed in the analysis of soluble solids content and reducing sugars, where both increased with the addition of the pomace.

**Table 1:** Physicochemical evaluation in samples with different grape pomace levels.

Components	Control	K25	K37.5	K50
Moisture (%)	97.21 ± 0.03a	95.56 ± 0.11b	95.51 ± 0.09b	94.82 ± 0.10c
Ash (%)	0.05 ± 0.04c	0.19 ± 0.05b	0.25 ± 0.05ab	0.31 ± 0.07a
pH	4.3 ± 0.03a	3.9 ± 0.03b	3.8 ± 0.03c	3.7 ± 0.03d
Titratable acidity (%)	2.07 ± 0.33d	3.0 ± 0.33c	4.9 ± 0.33b	5.9 ± 0.33a
Soluble Solids (° Brix)	2.5 ± 0.122c	3.9 ± 0.122b	4.2 ± 0.122a	3.9 ± 0.122b
Reducing Sugar (g/L)	4.96d	12.35c	21.18b	30.98a

Different letters in the row indicate statistical differences between means according to Tukey’s test (*p* < 0.05). Treatments tested: 0% (Control or KC), 25% (K25), 37.5% (K37.5), and 50% (K50) grape pomace addition.

Regarding the pH, we observed that the beverages exhibited values below 4.5. Furthermore, the beverages showed a reduction in pH value proportional to the increase in the concentration of added grape pomace, with the Control sample presenting a value of approximately 4.3 and the K50 sample a value of 3.7. It is important to highlight that beverages such as water-based kefir must have a low pH (typically between 3.0 and 4.2) to ensure product stability and safety, although this value is also determined by microbiological viability and sensory attributes. The acidity results corroborate those of the pH, where the addition of grape pomace is observed to increase the beverage’s acidity. This decrease in pH may also be associated with the production of organic acids, particularly lactic and acetic acids, during the fermentative process. In water-based kefir, lactic acid is typically the predominant organic acid formed by lactic acid bacteria through the metabolism of fermentable sugars, while acetic acid is produced in lower amounts via the oxidation of ethanol by acetic acid bacteria (34, 35).

The addition of grape pomace to water kefir significantly increased the total phenolic content (TPC), monomeric anthocyanins, and antioxidant activity (Table 2). A dose-dependent relationship was observed, with K50 exhibiting the highest TPC (4.31 ± 0.06 mg mL^−1^), while the control sample showed the lowest (0.74 ± 0.06 mg mL^−1^). Antioxidant capacity, measured by DPPH and FRAP assays, followed this same trend. Similarly, treatments K37.5 and K50 reached the highest monomeric anthocyanin concentrations (29.89 and 33.56 mg cyanidin-3-glucoside equivalent L^−1^, respectively), whereas these compounds were absent in the control. These findings align with the fact that approximately 60–70% of grape phenolic compounds remain in the pomace after vinification, representing nearly 5% of its dry matter. Our results corroborate previous studies identifying grape pomace as a primary source of bioactive compounds among agro-industrial residues, highlighting its potential for developing high-value functional beverages (36).

**Table 2:** Phenolic compounds (TPC), antioxidant activity (DPPH and FRAP), and monomeric anthocyanins.

Component	Grape pomace	Control	K25	K37.5	K50
TPC (mg/ml)	1.33 ± 0.03	0.75 ± 0.02d	1.51 ± 0.19c	2.54 ± 0.25b	3.13 ± 0.10a
DPPH (μmol Trolox Eq/mL)	17,760 ± 240	143.69 ± 18.1c	468.31 ± 14.3b	556.19 ± 12.7a	506.62 ± 37.9b
FRAP (μmol Fe^2+^/mL)	90,850 ± 325	109 ± 18.0c	2,555 ± 292.1b	8,480 ± 766.3a	8,496 ± 742.3a
Anthocyanins (cyanidin Eq, mg/L)	570.68 ± 25.37	0 ± 0c	16.83 ± 1.24b	14.81 ± 0.75b	21.8 ± 1.98a
Anthocyanins (pelargonidin Eq, mg/L)	661.19 ± 29.39	0c	19.51 ± 1.25b	17.15b	25.3a

Different letters in the row indicate statistical differences between means according to Tukey’s test (*p* < 0.05). Treatments tested: 0% (Control or KC), 25% (K25), 37.5% (K37.5), and 50% (K50) grape pomace addition.

This increase in TPC, anthocyanins, and antioxidant activity is a consequence of incorporating a substrate rich in phenolic compounds (grape skin) (3, 5) and the biotransforming action of the kefir microbiota during fermentation (4). Grape pomace, being a byproduct, acts as a dense matrix that retains significant quantities of polyphenols, many of which are bound to the cell wall (2). The introduction of this material substantially increases the initial concentration of Total Phenolic Compounds (TPC) in the beverage. Similar results were found in the study by Esatbeyoglu et al. (2), where samples prepared with aronia pomace (*Aronia melanocarpa*), for example, provided more monomeric anthocyanins (TAC) before and after fermentation when compared to the juice.

During the fermentation process, the symbiotic microbiota present in water kefir grains (including lactic acid bacteria) can induce the hydrolysis of complex phenolic compounds into lower molecular weight molecules (3, 33). This conversion is achieved by the activity of microbial enzymes, such as hydrolases and beta-glucosidases, resulting in the release of bound phenolic compounds and the formation of new antioxidant derivatives (5), as observed in our results.

Phenolic compounds and flavonoids are the main active substances that offer the capacity to scavenge free radicals (DPPH) and reduce transition metals (FRAP) (3, 4). Additionally, anthocyanins, pigments that are members of the flavonoid group, possess free radical scavenging properties and contribute to health benefits. Consequently, the increase of these compounds in the beverages explains the enhanced antioxidant capacity of the drinks. On the other hand, it is important to highlight that samples K37.5 and K50 were statistically similar in terms of antioxidant capacity as measured by FRAP; and the DPPH results for samples K25 and K50 were statistically similar (Table 2). This may be related to the lower pH of the K50 beverage, as well as having the highest content of fixed mineral residue (minerals), which in turn can form ions and are potentially complex with the antioxidant compounds, thereby reducing their activity.

### 3.2 Physical and chemical stability during storage

The variation in the pH of the beverage over time was significantly influenced (*p* < 0.05) by both the addition of grape pomace and the storage time (Table 3).

**Table 3:** ANOVA table for pH, soluble solids, acidity, anthocyanins and phenolic compounds during storage of kefir added grape pomace.

Components		pH	Acidity (% total acid)	Soluble solids	cianidina-3-glicosídeo Eq.mg/L	perlargonidina-3-glucosídeo Eq.mg/L	CFT
*R* ^2^		0.97	0.96	0.97	0.90	0.90	0.99
*F*		47.91	32.65	40.00	51.01	51.01	331.82
Pr > F		<0.0001	<0.0001	<0.0001	<0.0001	<0.0001	<0.0001
Grape pomace (%)	F	188.33	142.98	92.55	160.93	160.93	1025.88
	Pr > F	0.000	0.000	0.000	0.000	0.000	<0.0001
Time (Day)	F	38.23	10.32	85.19	22.38	22.38	168.58
	Pr > F	<0.0001	0.001	<0.0001	<0.0001	<0.0001	<0.0001
Grape pomace (%) × Time (Day)	F	4.33	3.32	7.42	5.60	5.60	39.21
	Pr > F	0.005	0.018	0.000	0.000	0.000	<0.0001

**Figure 1:**
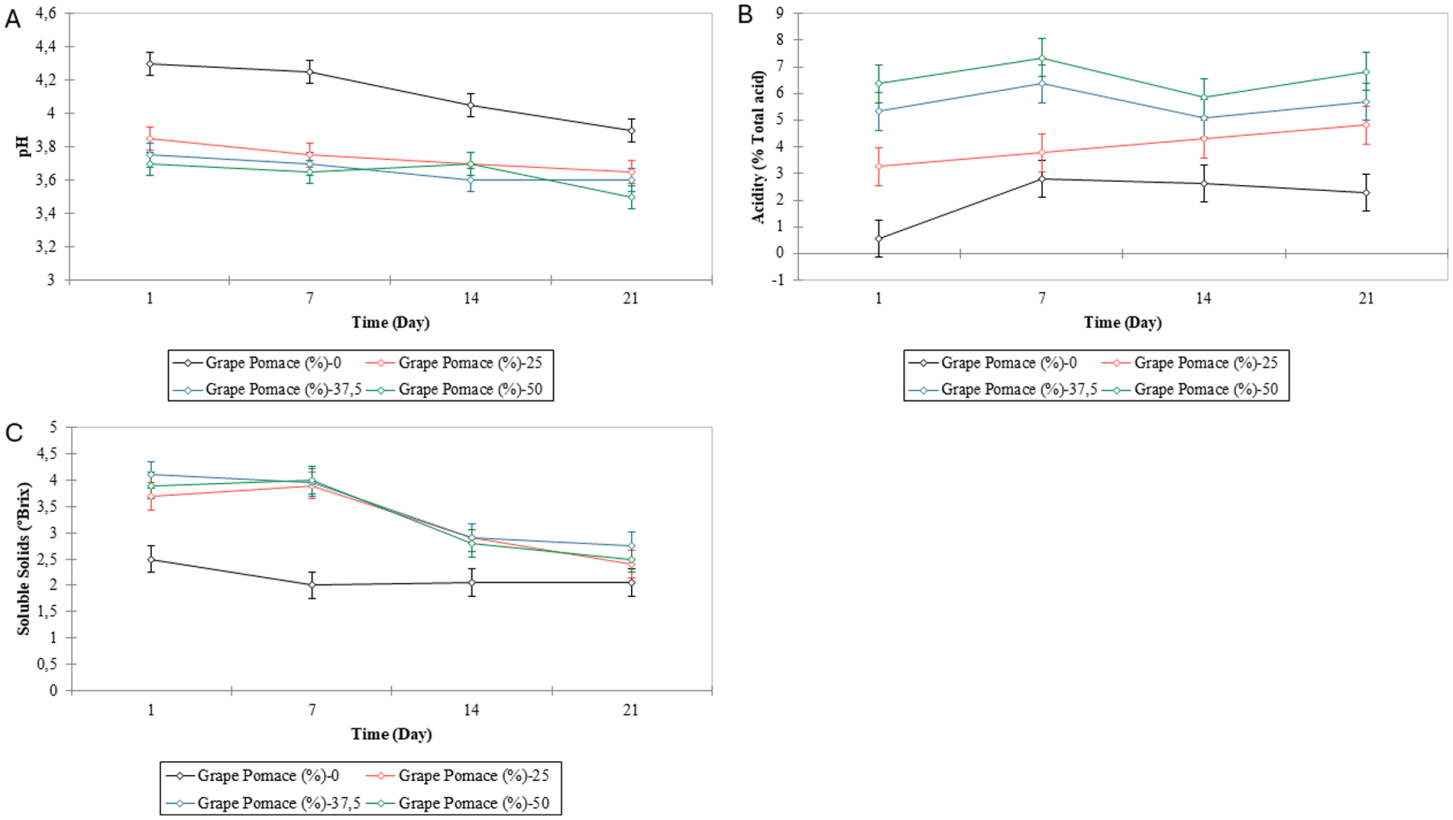
Evaluation of the titratable acidity of water kefir with added grape residue during refrigerated storage.

Both parameters reduced the pH of the beverage, i.e., the increase in the addition of pomace and the passage of storage time favored the reduction of kefir pH (control: 4.3 to 3.9; GPK25: 3.85 to 3.60; Figure 1A). The reduction in pH when grape pomace is added to water-based beverages, like kefir, is primarily due to the release of organic acids, polyphenols, and pectic acid, all of which contribute hydrogen ions to the solution, making it more acidic (37, 38). Additionally, the pH of water-based kefir with added grape pomace decreases during storage because ongoing microbial fermentation and the release of organic acids from the fruit both contribute to increased acidity. This process continues as long as the beverage is stored, resulting in a gradual drop in pH (37).

The results of total acidity in beverages during storage complement the results observed in the pH assessment, since samples with lower pH obtained higher total titratable acidity values (Figures 1A,B). Furthermore, the variables grape pomace concentration and storage time were directly proportional to the results obtained, i.e., beverages with higher grape pomace concentration were the most acidic, and the acidification process continued throughout storage (Table 3; Figure 1B). The acidity of drinks like grape pomace water kefir increases during storage because of ongoing microbial fermentation that produces organic acids from sugars and fruit components (37).

The control treatment had the lowest soluble solids content, with minimal variation during storage. On the other hand, the treatments with added grape pomace had higher soluble solids content. This is because grape pomace is rich in simple sugars (like glucose and fructose) in addition to the sugars already present in the base liquid. When fruit is added, the total amount of fermentable sugars increases significantly compared to kefir without fruit (3). It should also be noted that during storage, there was a reduction in soluble solids content, mainly in the first 7 days, indicating consumption of the remaining sugars in the beverage (Figure 1C). The reduction was greater in treatments with added grape pomace. Kefir with added fruit shows a greater reduction in soluble solids content during storage because the extra sugars from fruit fuel more active fermentation, leading to faster and more complete conversion of sugars into acids, alcohol, and other metabolites (3, 39).

**Figure 2:**
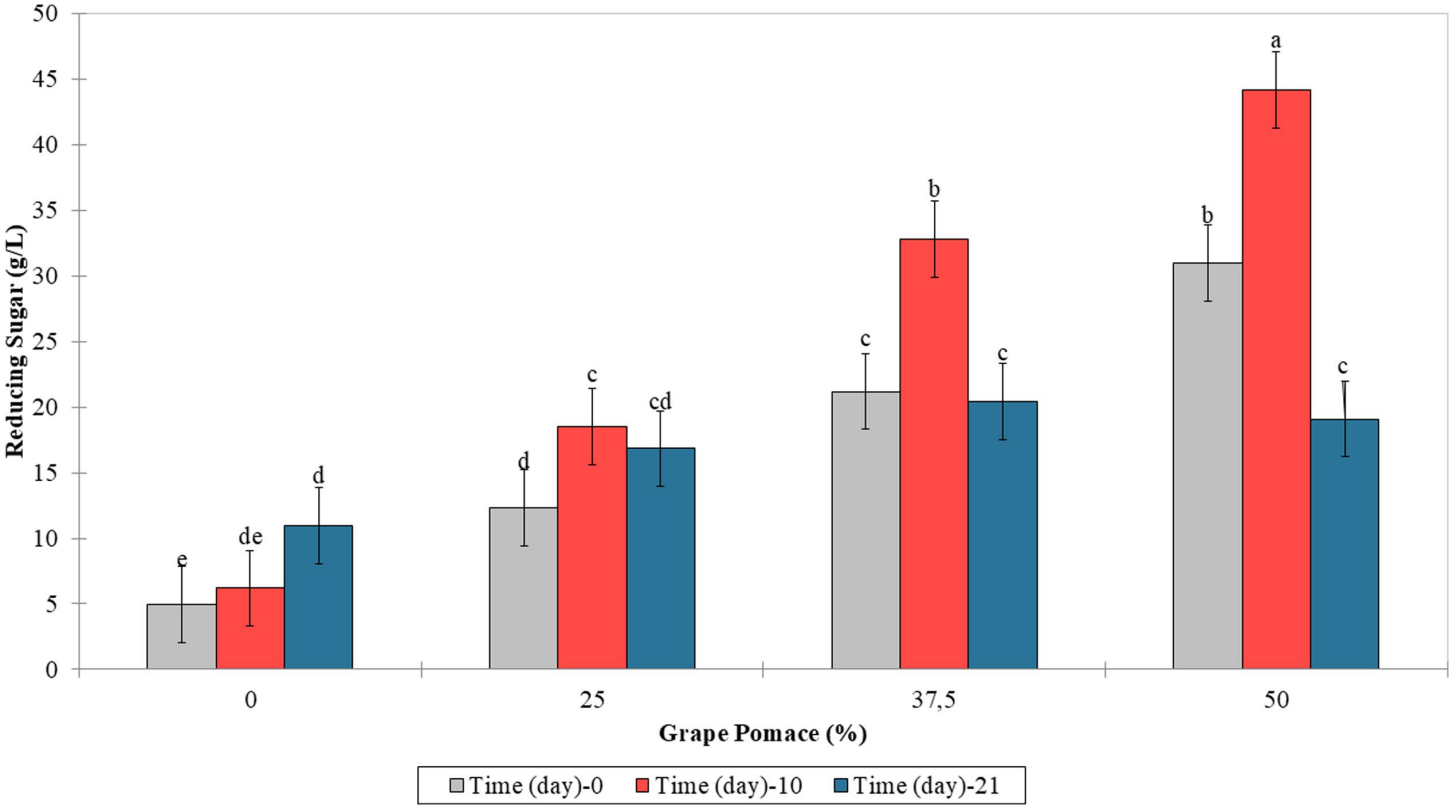
Evaluation of reducing sugars in water kefir supplemented with grape pomace during refrigerated storage. Different letters indicate statistical differences between means according to Tukey’s test (*p* < 0.05).

The results found in the evaluation of reducing sugar content throughout storage reaffirm the findings for pH, titratable acidity, and soluble solids, since beverages with added grape pomace showed an increase in reducing sugars followed by a reduction at the end of storage (Figure 2). This supports the hypothesis of the maintenance of microorganism metabolism during storage. Microorganisms, especially yeasts, rapidly hydrolyze sucrose into its monosaccharide components, glucose and fructose, both of which are reducing sugars. This enzymatic breakdown leads to a temporary rise in reducing sugar content as sucrose is converted faster than the microbes can immediately consume the resulting glucose and fructose (3). As storage continues, both lactic acid bacteria and yeasts begin to consume the available reducing sugars for their metabolic activities, converting them into organic acids, alcohol, and other metabolites. This leads to a reduction in reducing sugar content by the end of storage, as the rate of consumption overtakes the rate of sucrose hydrolysis (3, 39).

**Figure 3:**
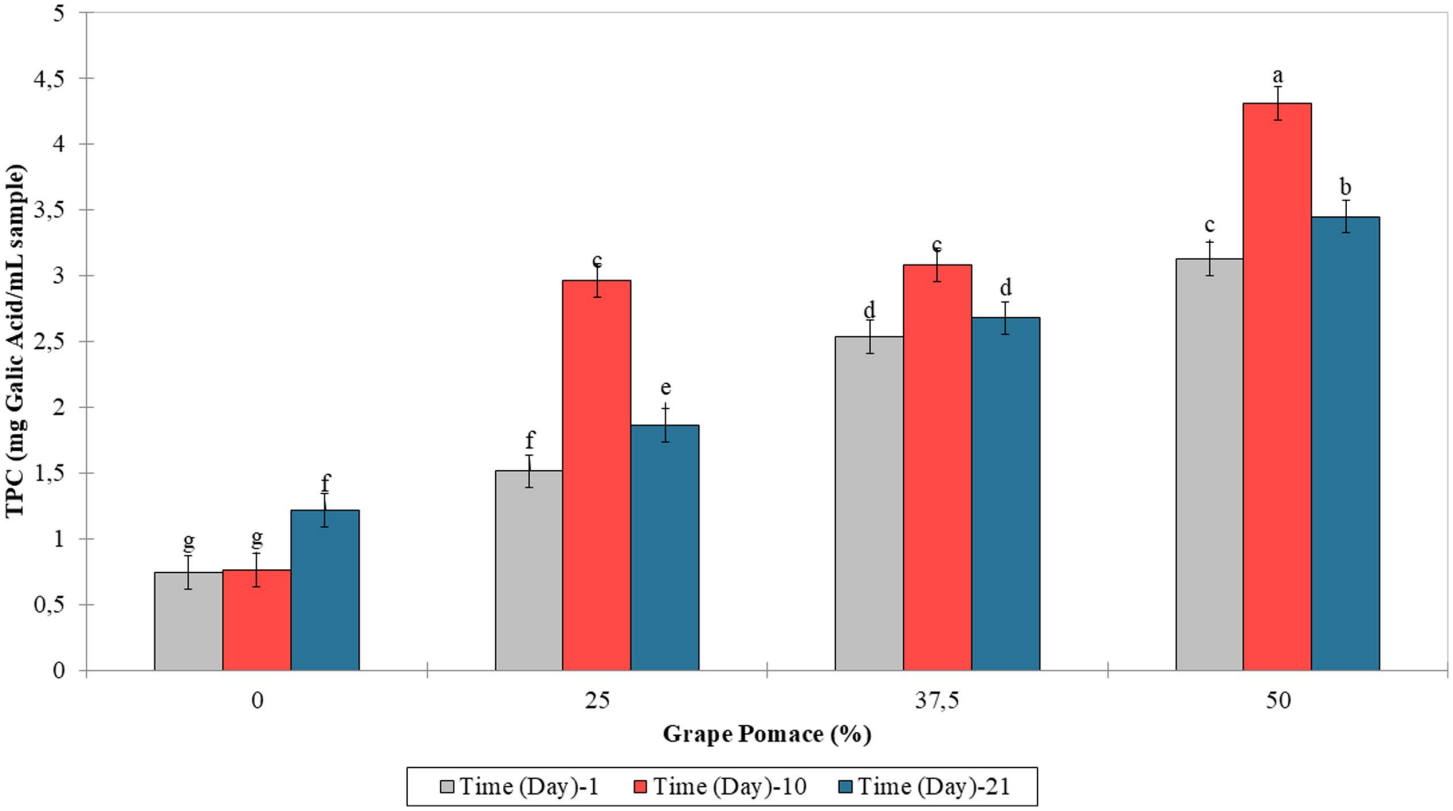
Evaluation of total phenolic compounds in water kefir supplemented with grape pomace during refrigerated storage. Different letters indicate statistical differences between means according to Tukey’s test (*p* < 0.05).

The phenolic compound content of fermented beverages showed similar behavior to that observed in the evaluation of reducing sugars, where both factors, grape pomace concentration and storage time, significantly (*p* < 0.05) influenced the results (Table 3). In other words, the samples with added grape pomace had the highest TPC levels, and over time, an increase followed by a decrease in TPC was observed (Figure 3). The increase in TPC in the first days of storage can be explained by microbial activity in the beverage. Microbial enzymes and acids break down fruit tissues and complex phenolic structures, releasing more free phenolic compounds into the beverage (6). Also, microbial metabolism can convert bound or complex phenolics into simpler, more detectable forms, temporarily raising the measured TPC (33). As storage continues, some phenolic compounds are degraded by microbial enzymes (like polyphenol oxidase) or oxidized, leading to a reduction in TPC. Additionally, phenolics can bind with proteins, peptides, or other matrix components, making them less extractable and thus lowering the measured TPC (6).

**Figure 4:**
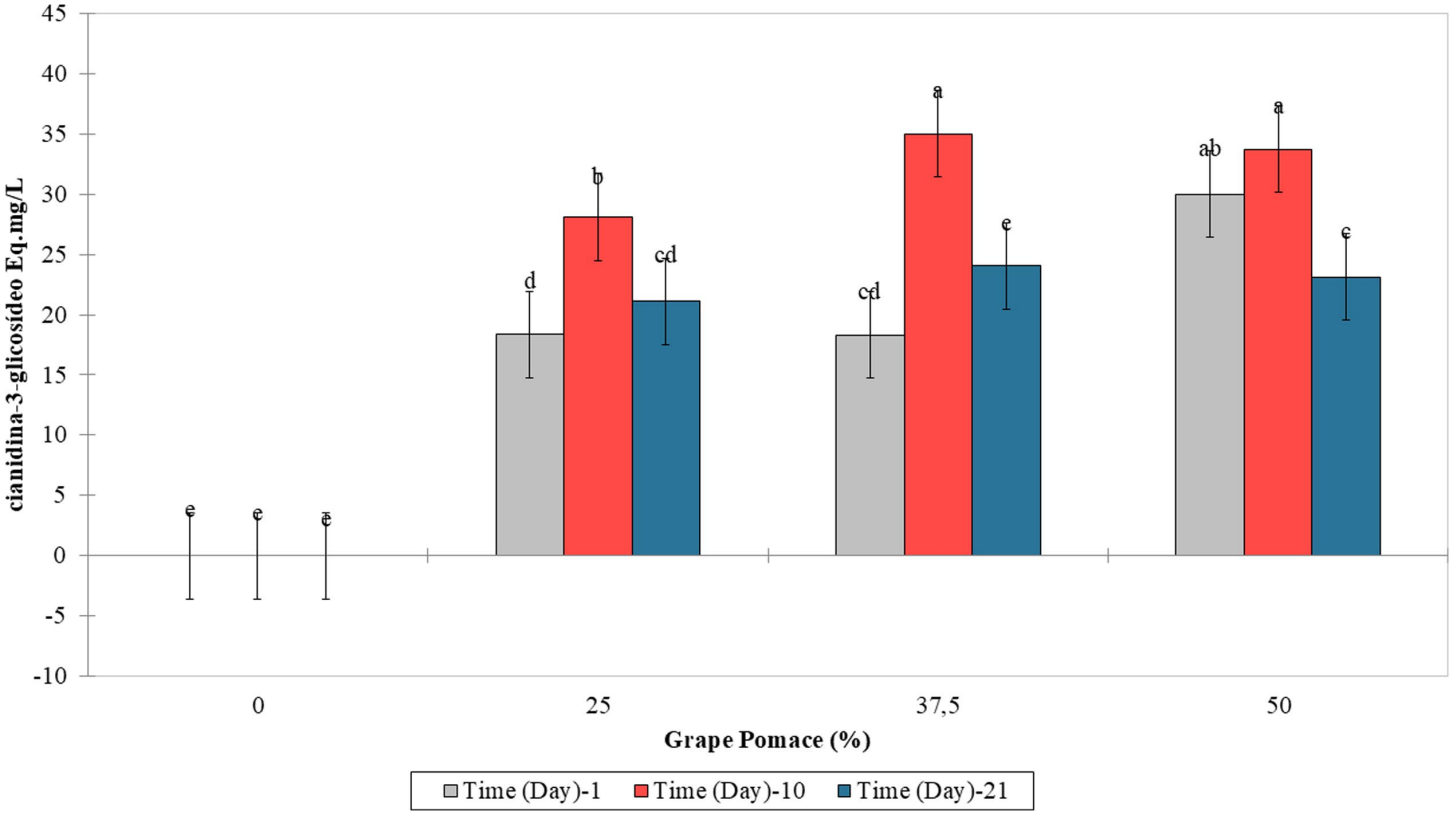
Evaluation of monomeric anthocyanins equivalent to cyanidin in water kefir supplemented with grape pomace during refrigerated storage. Different letters indicate statistical differences between the means according to Tukey’s test. (*p* < 0.05).

Like TPC, the monomeric anthocyanin content increased during the first 10 days of storage, and at the end of the storage period, there was a reduction in anthocyanin content. It should also be noted that the final value was statistically equal to the value on the first day of storage in samples with 25 and 37.5% grape pomace (Figure 4). Finally, it should be noted that the sample with the highest pomace content showed the greatest reduction (about 23%) in monomeric anthocyanins after 21 days of storage. According to Barazi and Arslan (6) in the first days of storage, anthocyanins are gradually extracted from fruit tissues into the kefir matrix, especially as microbial enzymes and acids break down cell walls and complex structures. This leads to a measurable increase in monomeric anthocyanin content. The ongoing fermentation process and acidification help solubilize anthocyanins, making them more available in the liquid phase. As storage continues, anthocyanins become less stable due to factors like low pH, enzymatic activity, and microbial metabolism. These conditions promote degradation, polymerization, and color changes, leading to a reduction in monomeric anthocyanin content (6). This explains the greater reduction in anthocyanin content in sample K50, since it had the lowest pH and highest acidity at the end of storage (Figure 2). Also, anthocyanins may also bind with proteins or other phenolic compounds, or undergo polymerization, making them less detectable as monomeric forms (6).

**Figure 5:**
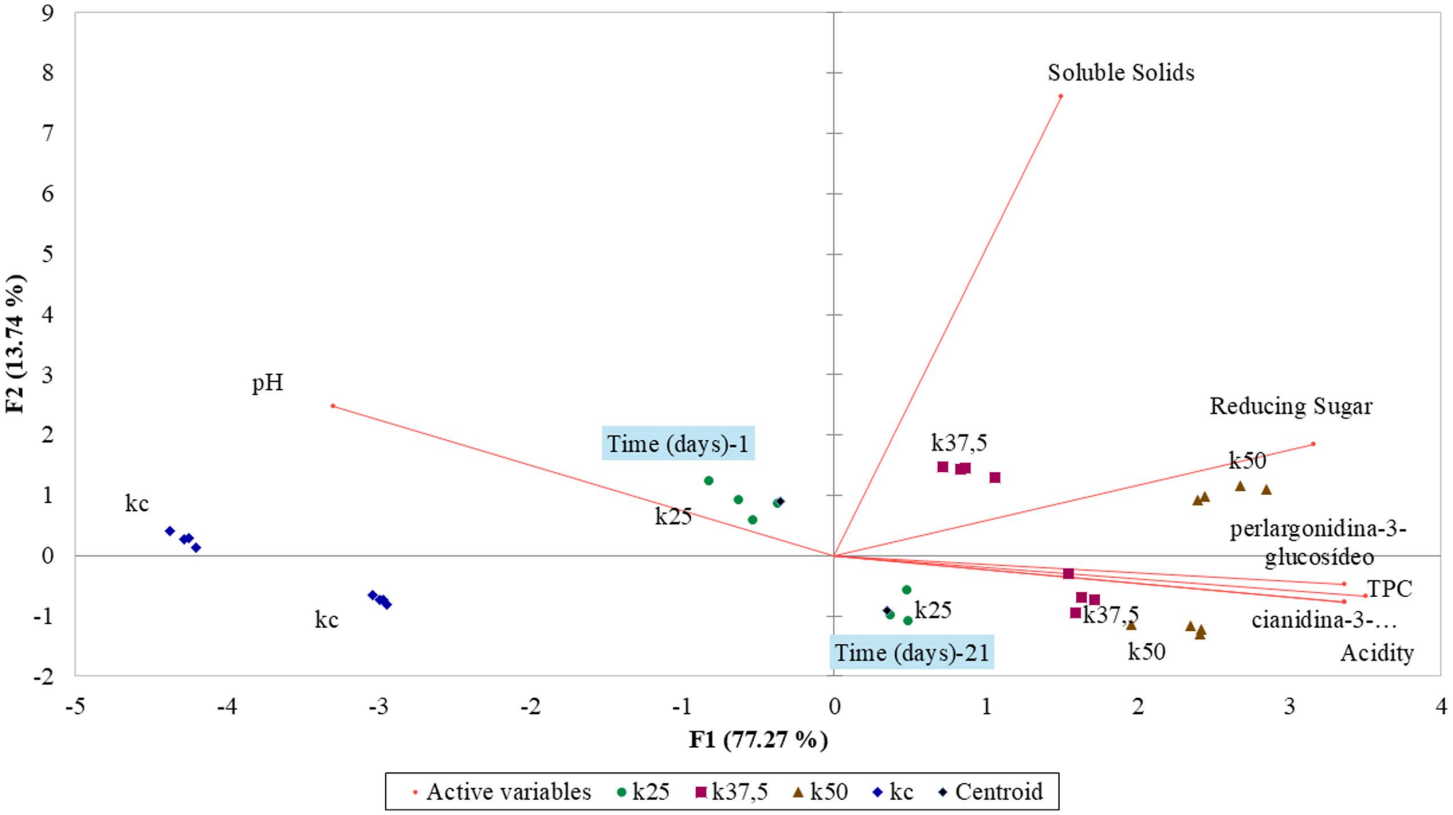
Principal component analysis (PCA) of the physicochemical properties of control kefir samples (KC) and samples with added grape pomace (K25, K37.5, and K50), as a function of storage time.

Principal component analysis highlights the effect of residue concentration in the beverage and time over the storage period, with 91.01% of the variability in responses explained (Figure 5). It should be noted that separation by F1 (77.27%) relates beverages with higher concentrations of grape pomace to higher values of anthocyanins, phenolic compounds, and reducing sugars. Furthermore, samples with 37.5% (K37.5) and 50% (K50) pomace show similar results at the end of storage, with high acidity being particularly noteworthy. It is also worth noting the pH, which decreased dramatically with increasing concentration. The high acidity of the beverage, combined with the presence of a high concentration of grape seed particles in the grape pomace, gave the beverage an astringent aftertaste, which did not please the tasters in sensory pre-tests (data not shown). The sensory acceptance test conducted in the study by Kesler et al. (40) showed that the most prevalent response to justify why tasters did not like commercial kefir was that it was sour. In other words, acidity is an important contributor to the acceptance of kefir. Given this, we concluded that the addition of 37.5 and 50% grape pomace (with seeds) is not feasible due to high acidity and residual taste. As a result, the beverage prepared with 25% (K25) grape pomace was considered the best and tested for functionality test in *C. elegans*, microbiological viability and sensory acceptance.

### 3.3 Functionality test

#### 3.3.1 Survival under thermal and oxidative stress, and longevity analysis

**Figure 6:**
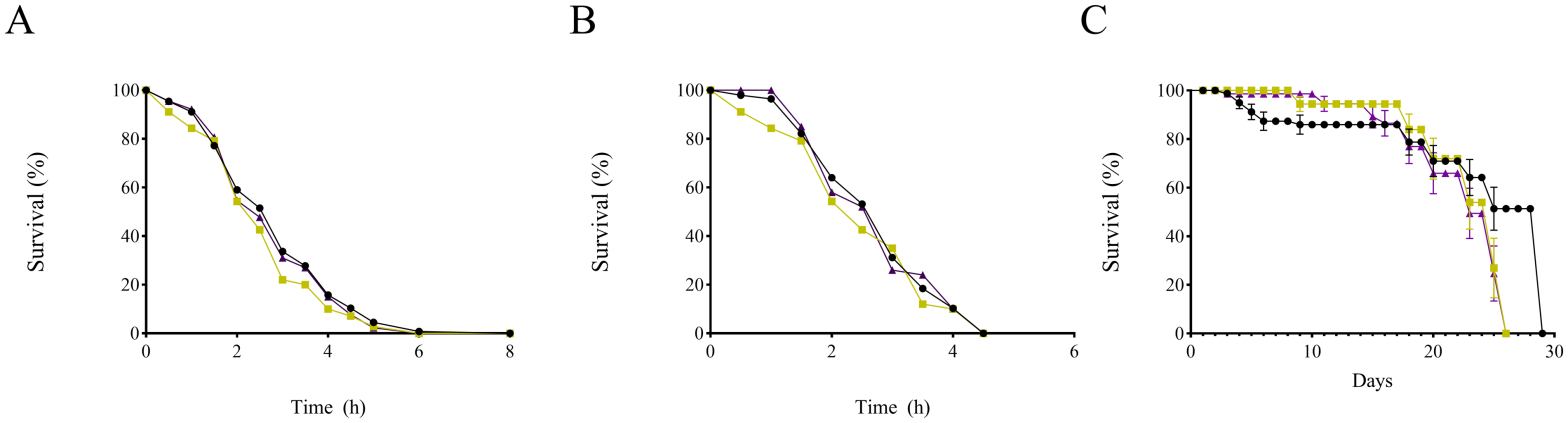
*Caenorhabditis elegans* survival. **(A)** Survival of *C. elegans* N2 (in hours) following H_2_O_2_-induced oxidative stress. **(B)** Survival of *C. elegans* BA17 (in hours) following heat shock at 35 °C; and **(C)** Survival of *C. elegans* BA17 (in days). (▲) K25, (■) KC, (●) K medium (control).

Exposure to K25 and Control Kefir beverages did not exhibit deleterious effects on the animals’ resistance to oxidative or thermal stress. Survival curves under oxidative stress did not differ among the groups (Log-rank Mantel–Cox, *p* = 0.2672; Figure 6A). Similarly, under thermal stress, the treatments displayed equivalent survival profiles (*p* = 0.1722, Log-rank Mantel–Cox; Figure 6B). These findings indicate that, although the treatments did not enhance the animals’ tolerance to the evaluated stressors (exposure to H_2_O_2_ and heat shock at 35 °C), neither beverage compromised the organisms’ capacity to cope with oxidative or thermal stress.

Survival analysis demonstrated that exposure to the K25 beverage did not significantly alter the longevity of *C. elegans* BA17. Survival curves for the group treated with the K25 beverage were similar to those of the control group (K medium) and the control beverage (KC), with mean lifespans of 25 ± 1.3, 28 ± 2, and 25 ± 0.5 days, respectively. The survival of animals maintained on Kefir K25 was statistically comparable to the survival curves of the controls: K medium and Control Kefir (Log-rank Mantel–Cox test, *p* = 0.1904). These results indicate that the K25 beverage exerts no negative effect on nematode lifespan under the evaluated experimental conditions (Figure 6C). Although diverse probiotics and fermented foods are capable of extending *C. elegans* lifespan by activating pathways such as DAF-16/FOXO and p38-MAPK/SKN-1 (25), K25 treatment did not promote longevity extension. This may reflect an absence of bioactive compounds capable of modulating these pathways, or methodological limitations regarding dosage, exposure time, or the sensitivity of the BA17 strain. Similar results, where specific fermented foods do not affect longevity, are reported in the literature (41).

Despite the absence of a geroprotective effect, our results are relevant for product safety, indicating that the K25 beverage does not compromise essential survival processes. Future studies may investigate additional health parameters, such as stress resistance, motility, and longevity gene expression, to determine whether K25 exerts subtle beneficial effects that are not directly reflected in lifespan extension.

#### 3.3.2 Fat accumulation in *Caenorhabditis elegans*

**Figure 7:**
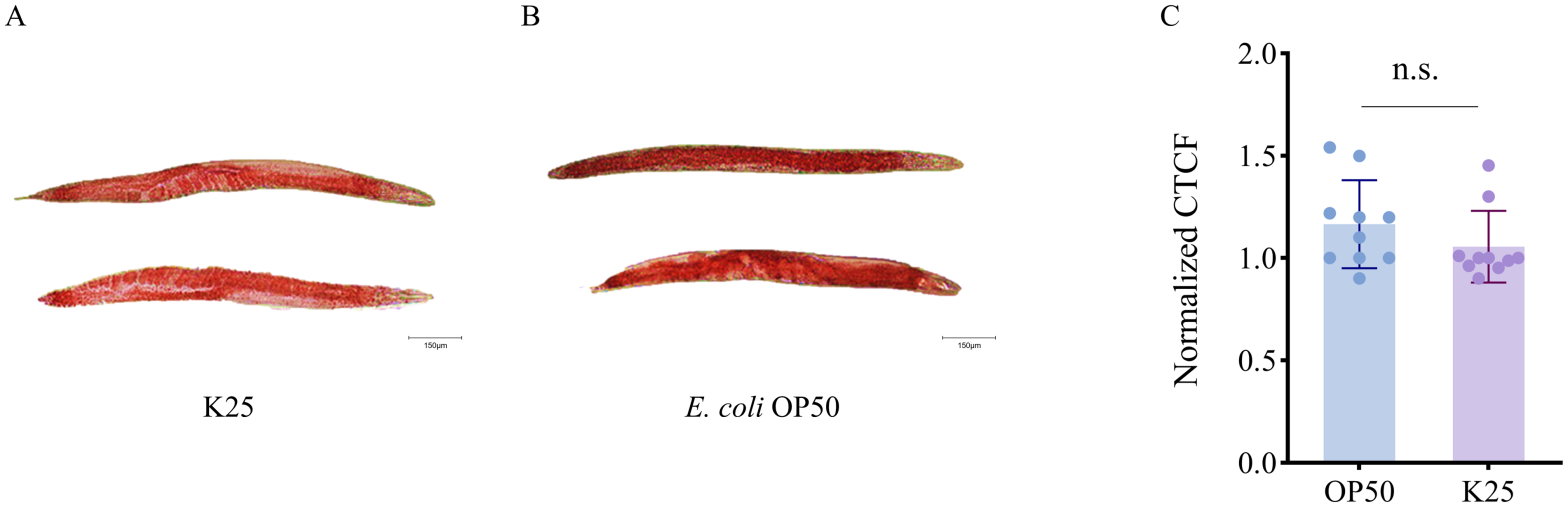
Representative images of lipid droplet staining. **(A)** Worms exposed to the K25 beverage stained with Oil Red O; **(B)** Worms from the *E. coli* OP50 control group; and **(C)** Quantification of lipid accumulation by Oil Red O staining.

Exposure of *C. elegans* to the K25 beverage did not significantly alter lipid accumulation compared to the control group fed *E. coli* OP50 (Figure 7). Quantification of normalized fluorescence (CTCF) obtained via Oil Red O staining revealed similar values between groups, with no statistical difference observed (unpaired *t*-test, *p* > 0.05). This result supports the conclusion that the K25 beverage does not negatively affect the lipid balance of this model organism, a relevant finding regarding metabolic safety (41, 42). Complementary investigations, such as gene expression analyses (e.g., *fat-6*, *fat-7*, *sbp-1*, *aak-2*) or assays under metabolic stress conditions, may more accurately elucidate whether the product possesses subtle effects not evidenced by the basal lipid accumulation analysis.

### 3.4 Microbiological viability during storage

The study of the microbiological viability of acetic acid bacteria, yeasts population, and lactic acid bacteria demonstrated that, during storage, the acetic acid bacteria population remained stable, while an increase in yeast and lactic acid bacteria populations was observed (Table 4).

**Table 4:** Counts of acetic acid bacteria (AAB), yeasts, and lactic acid bacteria (LAB) in control Kefir (KC) and K25 samples during storage.

Time (day)	1	7	14	21
Acetic acid bacteria (AAB) (CFU/mL)				
KC	8.0 × 10^5^	2.45 × 10^6^	1.05 × 10^6^	1.25 × 10^6^
K-25	8.05 × 10^7^	1.3 × 10^7^	1.8 × 10^7^	3.8 × 10^7^
Yeast (CFU/mL)				
KC	4.0 × 10^4^	2.05 × 10^6^	2.05 × 10^5^	2.45 × 10^5^
K-25	2.1 × 10^7^	3.65 × 10^6^	1.45 × 10^5^	1.0×10^7^
Lactic acid bacteria (LAB) (CFU/mL)				
KC	—	—	5.0 × 10^4^	1.3 × 10^5^
K-25	5.0 × 10^4^	2.25 × 10^4^	7.0 × 10^4^	1.2×10^5^

Treatments tested: 0% (Control or KC) and 25% (K25) grape pomace addition.

The rich matrix introduced by the grape pomace alters the fermentation ecosystem. This environment selectively favors the growth and sustains high viability of acid-tolerant acetic acid bacteria (AAB). The initial reduction in pH observed upon pomace addition (K 50 reached 3.7) establishes a highly selective environment, primarily favoring the growth of acid-tolerant acetic acid bacteria (AAB). Consequently, the AAB component becomes the primary driver of the final physicochemical characteristics, utilizing ethanol and lactic acid to produce acetic acid. This metabolic activity explains the consistently low pH (ranging from 3.5 to 4.3 during storage) and the significant increase in total titratable acidity (up to 6.60% of total acid), overriding the expected metabolic equilibrium of traditional water kefir fermentation by prioritizing acetous fermentation (43–45).

Conversely, the substrate selectively challenges the viability of the yeast and lactic acid bacteria (LAB) populations. The restriction of LAB growth relative to control is strongly linked to the antimicrobial activity of the phenolic compounds and anthocyanins extracted from the grape residue. These bioactive components exert a selective inhibitory action on sensitive microorganisms, thus challenging LAB viability. Additionally, LAB strains face intensified competition with the highly vigorous yeast population for fermentable sugars (glucose and fructose) present in the pomace-enriched medium (3, 45).

The viability of the yeast population itself is volatile. While yeast benefits from the initial sugar enrichment provided by the pomace, leading to a high initial count, the subsequent reduction observed during refrigerated storage is likely due to the cumulative effects of metabolite toxicity, specifically the accumulating acetic acid produced by AAB and the increasing ethanol content, and the continued antimicrobial potential of the concentrated phenolic compounds. The slight later recovery (as seen at day 21) suggests the presence of a more complex, slowly hydrolysable nutrient released from the residual grape pomace matrix over time, allowing them to resume growth and metabolic activity (46). While water kefir consortia are typically characterized by yeasts from the genera *Saccharomyces, Zygosaccharomyces*, or non-Saccharomyces strains, further molecular characterization is required in future studies to determine the exact taxonomic shifts induced by the grape pomace.

### 3.5 Sensory acceptance and intention to purchase

Microbiological safety assessments confirmed that all samples met current Brazilian legislation standards, showing an absence of *Salmonella*/25 mL and counts < 10 CFU mL^−1^ for *E. coli*, molds, and yeasts. Consequently, the fermented beverages were considered safe for sensory evaluation. One hundred and twenty untrained tasters (*n* = 120) participated in the sensory analysis. Most of them reported that they do not habitually consume non-alcoholic fermented beverages (58.33%). On the other hand, 35.83% stated that they consume fermented beverages less than once a week, 1.66% once a week, 2.5% three to four times a week, and 1.66% stated that they consume them once a day. These results are justified by the preferences of Western consumers, who prefer mild, sweet, and low-acid flavors, while fermented beverages differ in that they have more complex, acidic, or bitter flavors. The study by Walsh et al. (47) pointed out that traditional and commercial kefir has limited acceptance due to its strong acidity, unusual flavor, and viscous texture. In addition, fermentation also gives it a distinct fermented aroma resulting from volatile acids, aldehydes, esters, ketones, and sulfur compounds.

**Figure 8:**
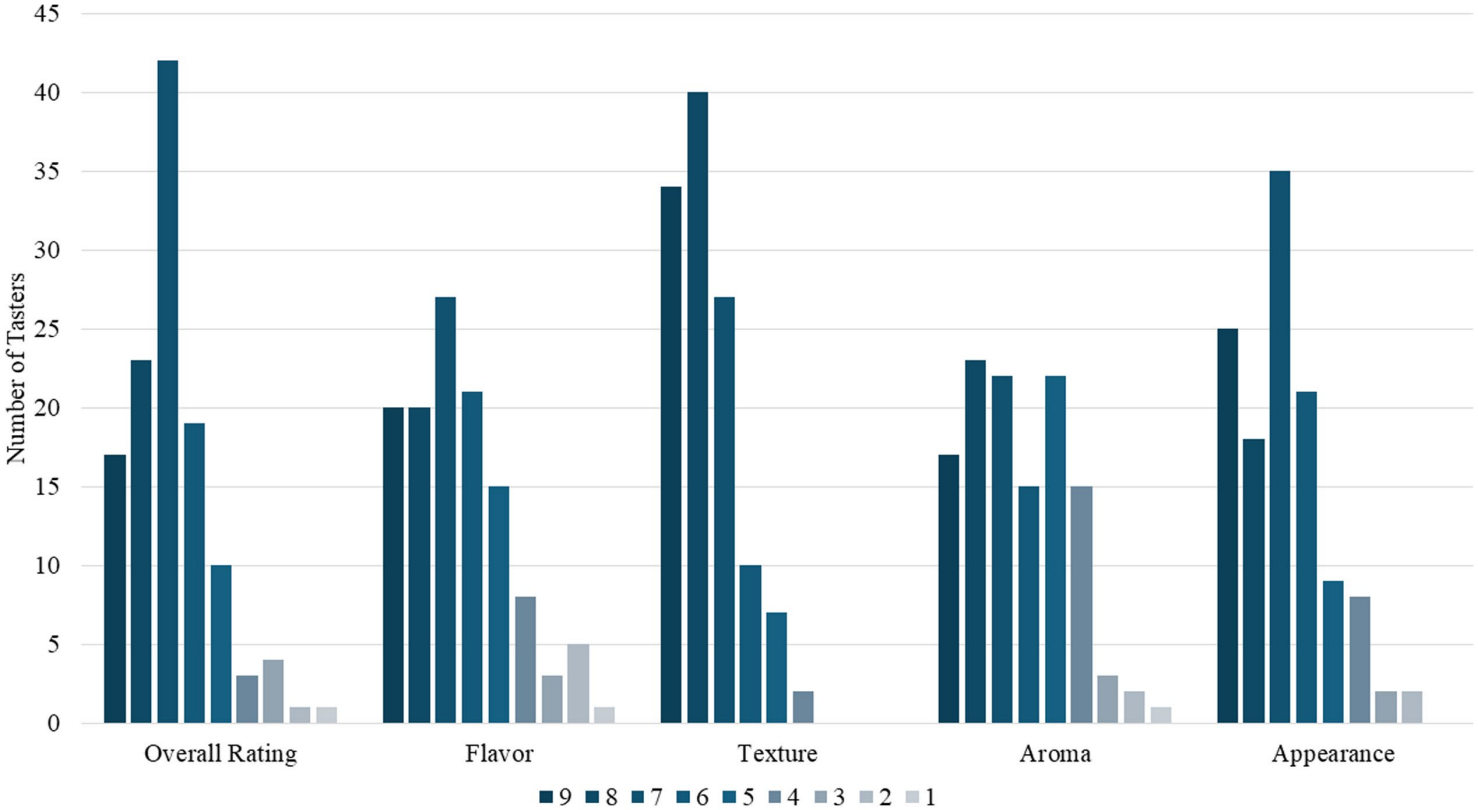
Frequency of scores (9-point hedonic scale, where 9 = I liked it very much, 5 = I neither liked nor disliked it, and 1 = I disliked it very much) assigned for appearance, color, aroma, flavor, texture, and overall evaluation of water kefir flavored with grape pomace (wine-making waste). *n* = 120.

**Figure 9:**
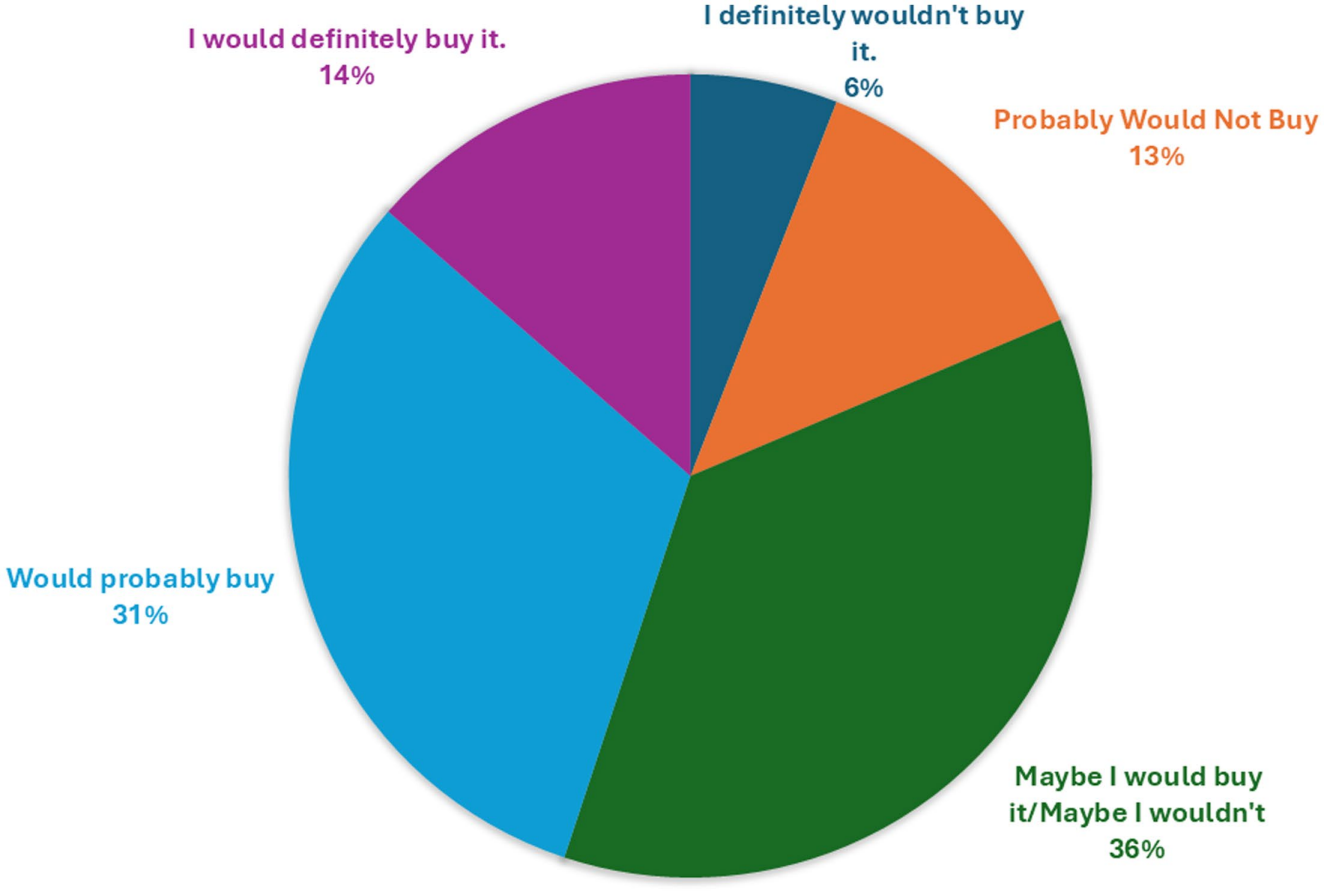
Intention to purchase kefir with 25% grape pomace (K25). *n* = 120.

The overall acceptance rate was 76.11%, with an average score of 6.85 ± 1.58, where 33% of the tasters reported liking the drink very much or extremely much (Figure 8). For the attributes of flavor, texture, aroma, and appearance, the average scores obtained were 6.52 ± 1.91, 7.65 ± 1.23, 6.38 ± 1.87, and 6.89 ± 1.66, respectively. Of these, the high acceptance of the drink’s texture stands out, with 62% of tasters indicating that they liked this attribute of the drink very much or extremely (Figure 8). This positive result can be explained by the grape flavor presented by kefir, after all, the fruit and its by-products, such as juices and wines, are well accepted due to various sensory and cultural factors associated with fruit. Thus, although most consumers are not in the habit of consuming kefir or other non-alcoholic fermented beverages, the product developed with 25% grape pomace was well accepted sensorially. As for the results of the intention to purchase kefir with added grape pomace, the most frequent response was “unsure whether I would buy it” (35.8%), followed by “I would probably buy it” (30%), and “I would definitely buy it” (13.3%). Thus, 43.3% of tasters expressed an intention to purchase the drink if it were available for sale on the market. Less than 20% indicated that they had no intention of purchasing the drink (Figure 9).

From a practical and industrial perspective, the findings of this study offer a dual benefit for sustainable food development and waste valorization. First, this research demonstrates that grape pomace can be successfully up-cycled without intensive chemical modification, providing the wine industry with a high-value alternative to reduce agro-industrial waste disposal costs. Second, for the functional beverage sector, the K25 formulation establishes a clear technological baseline for the formulation of non-dairy, plant-based probiotic carriers that align with current consumer demands for clean-label, bio-inclusive, and antioxidant-rich products.

## 4 Conclusion

The incorporation of grape pomace significantly alters the water kefir fermentation ecosystem, increasing the concentration of bioactive compounds—such as phenolics and anthocyanins—and enhancing antioxidant activity. Higher concentrations (37.5 and 50%) led to intense acidification and lower stability; thus, the 25% formulation (K25) was identified as the optimal dosage, maintaining high microbial counts (10^7^ CFU mL^−1^). The grape pomace acted as a selective driver of the microbial successional pathway, favoring acid-tolerant acetic acid bacteria (AAB) while challenging the viability of lactic acid bacteria (LAB) and yeast. Regarding biological safety, the K25 beverage did not compromise essential survival processes or alter lipid accumulation in the *Caenorhabditis elegans* model. Although no geroprotective effects were observed under basal conditions, these results confirm the metabolic safety of the product. Sensory analysis revealed that K25 was well-accepted (76%), even by consumers unaccustomed to non-alcoholic fermented beverages, demonstrating its commercial viability.

While the use of a single-source residue constitutes a limitation, this study establishes a foundation for using winery by-products in probiotic carriers. Although the developed water kefir beverage demonstrated promising functional and sensory characteristics, achieving complete product standardization regarding shelf life and alcohol content control remains a critical prerequisite for commercial scalability. The inherent metabolic dynamism of the symbiotic culture of bacteria and yeast poses challenges for industrial-scale stabilization, as continuous post-bottling fermentation can alter both the volatile profile and ethanol concentrations over time. To bridge these technological gaps, future research must evaluate the seasonal stability and chemical consistency of the grape pomace, ensuring uniform fermentation kinetics across different production batches. Furthermore, while the *in vivo Caenorhabditis elegans* model confirmed the biological safety of the K25 formulation under basal conditions, subsequent studies should investigate the post-digestive bioaccessibility and bioavailability of its phenolic compounds using *in vitro* gastrointestinal digestion models. Investigating these parameters, along with the expression of key metabolic genes (e.g., *fat-7, sbp-1, aak-2*) under oxidative stress, will provide the necessary framework to establish a fully standardized, shelf-stable, and commercially viable probiotic carrier.

## Data Availability

The raw data supporting the conclusions of this article will be made available by the authors, without undue reservation.
